# Understanding Zika virus pathogenesis: an interview with Catherine Spong

**DOI:** 10.1186/s12916-016-0628-0

**Published:** 2016-06-06

**Authors:** Catherine Y. Spong

**Affiliations:** Eunice Kennedy Shriver, National Institute of Child Health and Human Development, National Institutes of Health, Bethesda, MD USA

**Keywords:** Zika, Infants, Pregnancy, Neurology, Microcephaly, Miscarriage, Stillbirth, Infectious disease, Cohort, Fetal anomaly

## Abstract

A recent outbreak of Zika virus has been linked to fetal abnormalities in pregnant women who have been infected. The scientific community is working toward understanding Zika virus pathogenesis to better manage affected women and children. In an interview with Dr. Catherine Spong, we discuss the aims and challenges of a forthcoming longitudinal study of a cohort of pregnant women in areas of current active Zika virus transmission.

## Introduction

**Fig. 1:**
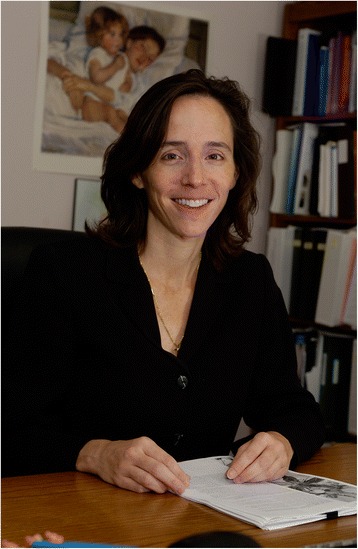
Catherine Y. Spong MD

Dr. Catherine Spong (Fig. 1) is the Acting Director of the *Eunice Kennedy Shriver* National Institute of Child Health and Human Development at the National Institutes of Health. In this role, she oversees research on pediatric health and development, maternal and reproductive health, intellectual and developmental disabilities, and rehabilitation medicine, among other areas. Her areas of expertise include maternal and child health, particularly prematurity, fetal complications, and improvement of child health outcomes. Her Institute is currently recruiting for a multicenter, longitudinal cohort study looking at the effects of Zika in infants and pregnancy.

## **1.** What do we know so far about Zika virus infection and pregnancy outcomes?

Zika virus infection in pregnancy has been linked to adverse complications, including miscarriage, stillbirth, fetal brain anomalies, and eye disorders. Many questions about prenatal Zika virus infection remain unanswered. With the information currently available, we are unable to inform a woman about the risk to her pregnancy if she is infected with the Zika virus. We have little information on how often prenatal Zika infection results in adverse outcomes such as miscarriage or microcephaly. The current literature is populated mostly by studies that followed women who had symptoms of Zika virus infection, such as rash or fever. Given that an estimated 80 % of people with Zika virus infection have no symptoms, we need to conduct studies in this population to understand the risk of asymptomatic Zika virus infection among pregnant women. We know that in other infections in pregnancy, the timing of the infection will help determine the outcome. The first trimester (weeks 0–13) is commonly thought to be the most vulnerable period because that is when most of the organ formation occurs. Some viral infections cause the most damage when the infection occurs during this time period. However, studies of symptomatic pregnant women with Zika virus have found that infection even in the second or third trimesters has resulted in anomalies in the fetal brain and other serious outcomes, including stillbirth, which is very concerning.

## **2.** How has NIH responded to this public health emergency?

The National Institutes of Health (NIH) is very interested in studying Zika virus and has published two notices (NOT-AI-16-026 and NOT-HD-16-004) highlighting the critical areas for research. Notably, we have launched a rapid solicitation (PAR-16-106) for R21 applications that allows submissions without preliminary data. The solicitation opened on March 20, 2016, and applications are being accepted on a rolling basis. In my over 20 years of experience at NICHD, this is the first time I have seen the use of the rapid funding opportunity announcement, which is a mechanism that enables continuous receipt and review of applications and then expedited funding. Eight NIH institutes and centers have signed on to this opportunity: NICHD, the National Institute of Allergy and Infectious Diseases, the National Institute of Dental and Craniofacial Research, the National Institute of Neurological Disorders and Stroke, the National Institute of Mental Health, the National Eye Institute, the National Institute of Biomedical Imaging, the National Heart, Lung, and Blood Institute, and the NIH Division of Program Coordination, Planning, and Strategic Initiatives. Other institutes are likely to join given the multifocal nature of this public health emergency.

NIH’s interest includes basic research to understand Zika infection pathogenesis and transmission to the fetus, establishment of animal models to help translate laboratory findings into living systems, developing diagnostics and vaccines, and clinical studies. This clinical work includes understanding the role of Zika infection in pregnancy complications, as well as studies of children and non-pregnant adults to evaluate neurologic complications such as Guillain–Barre syndrome. I encourage wide dissemination of this funding opportunity to the scientific community.

## **3.** What areas of study do you find most important in the context of Zika?

We plan to study pregnant women in regions where the virus is spreading to discern the risk of adverse outcomes to a woman infected during her pregnancy, whether she is symptomatic or not. We also want to establish the impact that the timing of infection has on pregnancy outcomes and the role, if any, of prior exposures to other pathogens, such as dengue virus, and to environmental factors such as pesticides, larvicides, and insect repellents.

Additionally, we want to document the spectrum of neonatal outcomes related to fetal exposure to Zika virus infection. This includes not only microcephaly, which has been reported extensively since the outbreak began, but also other serious brain anomalies, fetal loss/stillbirth, eye abnormalities, and developmental and motor outcomes. It is likely that microcephaly is one of the severe complications and that more subtle anomalies will be identified once systematic studies are performed.

## **4.** Who are you recruiting?

We aim to recruit approximately 10,000 pregnant women at up to 15 sites across Latin America and the Caribbean, regions currently experiencing active Zika virus transmission. We anticipate starting recruitment in early summer. The participants will be in their first trimester, and we will follow them to determine timing of exposure to Zika virus and whether the women become symptomatic or not. We will use CDC testing kits to detect Zika infection. However, if new, validated diagnostic tests become available, we will systematically incorporate them into the study.

We also want to study the presence of potential confounding factors, such as prior infections, including dengue and cytomegalovirus, and environmental exposures to determine what role they may play in the severity of maternal or infant disease. Our preference is to have patients enroll as early as possible in pregnancy because this will improve our ability to evaluate the impact of the timing of infection on pregnancy outcome.

## **5.** How are you recruiting?

Recruitment will occur through prenatal clinics and other community outreach, depending on the study site, region, and country. We recognize that there are different cultures and methods of providing healthcare globally, and we will work with local groups to determine the optimal method for recruitment.

For each site, we will obtain ethics approvals and participant consent in accordance with local practices. To participate in this study, all sites will be using the same methods to gather information and materials, and the data will be sent to a central location for monitoring and analysis.

## **6.** How long do you intend to follow the cohort?

We intend to monitor the women for the duration of their pregnancies and for 6 weeks after they give birth. We will follow the infants for up to 2 years after birth. It will be important for us to follow the children and mothers after delivery to determine the impact of prenatal Zika virus infection and to provide sufficient assessment to document the outcomes. This follow-up will require clinical examinations, eye exams, imaging studies, and the collection and analysis of biospecimens.

## **7.** What are the challenges?

Clinical research is inherently challenging. For this study, we will be challenged by our ability to diagnose women who are infected with Zika virus – but are asymptomatic – in areas where dengue virus is also endemic. The current diagnostic test for Zika cross-reacts with dengue virus, and therefore diagnosing Zika infection may be complicated. In addition, viremia evaluation, or the presence of virus in the blood, following Zika exposure may vary between asymptomatic and symptomatic women.

Some women may not want to participate or will decide to drop out of the study given the concerns and implications of a Zika diagnosis. Although all studies are concerned with loss to follow-up or “drop-out”, in this situation where the visibility is so high and the risks so personal, it is something we need to watch closely. Follow-up may be difficult, especially if children have no signs of abnormalities at birth.

For this study, it will be critically important to have follow-up of all the children, and not just those with complications at delivery, to determine the complication rate and to evaluate brain and developmental milestones. The concern with losing study participants is the ensuing difficulty of interpreting the study results.

One of the key components of this study is that all participating sites will use the same protocol, so the same information will be collected regardless of site or country. This is essential for us to be able to perform the analyses and to answer the critical questions. We have worked very closely with several other groups interested in studying the impact of Zika virus on pregnancy, and we have shared our protocol so that they too will collect information in the same way, enabling us to possibly collaborate in the future. In addition, we are working in concert with health agencies in affected countries.

## **8.** Is there anything that the wider scientific community can do to help with this study?

There has been significant outreach by the research community. Some groups are making their data available immediately. One example is the Zika Open Research portal of O’Connor and Osorio (https://zika.labkey.com/project/OConnor/ZIKV-001/begin.view). This allows the community to learn their findings immediately, provides opportunities for collaboration, and helps others to generate new research ideas.

We have been working with other groups on the upcoming cohort study. For example, Fiocruz, a research institution that is part of the Brazilian Ministry of Health (http://portal.fiocruz.br/en/content/home-ingl%C3%AAs), is partnering with NIH on the study by providing scientific expertise and co-funding some of the sites. We encourage other groups interested in launching similar studies to contact us, so we can share information and facilitate potential collaborations.

## **9.** In addition to getting more information to better manage Zika in pregnancy, are there other aims of this study?

We need to understand the full impact of the effects of Zika virus on pregnancy outcomes. A variety of fetal neurologic abnormalities, such as cerebellar hypoplasia (the infant’s cerebellum is reduced in size but normal in shape), global cerebral hypogyration (the folds of the brain, or gyri, are reduced in number and size), and intracranial calcifications (calcium deposits in brain tissue), have already been reported in these children, and we need a comprehensive evaluation of these cases. We also need to understand the spectrum of outcomes in asymptomatic women.

Further, we need better understanding of Zika virus-related eye and hearing abnormalities, as well as neurobehavioral effects such as altered motor function, stiffness, irritability, and incessant crying. It will be essential to follow the children after birth to evaluate their neurodevelopment and developmental milestones, including children who show no signs of anomalies, to fully characterize the impact of Zika on child health outcomes. Characterizing the full extent of Zika virus impact on fetal development will enable the development of strategies to assess and monitor the growth of children exposed to the virus in the womb and to define and optimize ways to treat and care for these children.

## **10.** Where can I find out more?

See reference list [1–10].
